# Mapping antibiotic resistance in Ghana: a narrative review of regional variations in antibiotic-resistant ESKAPEE pathogens

**DOI:** 10.3389/fmicb.2025.1696696

**Published:** 2026-01-06

**Authors:** Abdulwasid Abubakari, Rosemary Agbeko, George Osei-Adjei, Rolf Kümmerli

**Affiliations:** 1 Department of Science Laboratory Technology, Accra Technical University, Accra, Ghana; 2 Department of Biochemistry, School of Biological Sciences, College of Agriculture and Natural Sciences, University of Cape Coast, Cape Coast, Ghana; 3 Department of Quantitative Biomedicine, University of Zurich, Zurich, Switzerland

**Keywords:** antimicrobial resistance, ESKAPEE pathogens, Ghana, One Health, regional heterogeneity, surveillance

## Abstract

**Introduction:**

Antibiotic resistance (ABR) is a global health crisis and a significant public health challenge in Ghana. This narrative review aims to uncover the patterned landscape of ABR by systematically assessing regional heterogeneity across three key dimensions: (1) the geographic distribution and intensity of ABR surveillance and research; (2) the prevalence of ESKAPEE pathogens (*Enterococcus faecium*, *Staphylococcus aureus*, *Klebsiella pneumoniae*, *Acinetobacter baumannii*, *Pseudomonas aeruginosa*, *Enterobacter* species, and *Escherichia coli*); and (3) the distribution of their antimicrobial resistance profiles and underlying genetic mechanisms. The review maps data from clinical, animal, and environmental sources to provide a comprehensive sub-national picture.

**Methods:**

Four online databases (PubMed, Scopus, Web of Science, and African Journals Online) were searched for relevant articles published from 2016 to 2025, a timeframe chosen to align with the development and implementation of Ghana’s first National Action Plan (NAP) on AMR (2017–2021). A narrative synthesis was conducted to collate, analyze, and map regional data on ESKAPEE pathogen prevalence, resistance patterns, and underlying genetic mechanisms.

**Results:**

A total of 48 studies met the inclusion criteria. The findings reveal a significant disparity in research coverage, with a heavy concentration of studies in the Greater Accra (35.4%) and Ashanti (18.6%) regions. Widespread multidrug resistance was evident across all studied regions, with *E. coli* and *K. pneumoniae* consistently showing high resistance (>70%) to common antibiotics like ampicillin and third-generation cephalosporins. The review documents the emergence of critical carbapenemase genes (including *blaNDM-1* and *blaOXA-48*), a notable prevalence of methicillin-resistant *S. aureus* (MRSA), and the detection of the colistin resistance gene *mcr-1* in animal sources. Significant heterogeneity in study design and methodology was observed across the included literature.

**Conclusion:**

ABR in Ghana presents as a patterned landscape rather than a uniform national profile. Stark regional disparities and major surveillance gaps create “blind spots” that can hinder the effective implementation of national policy. Addressing this requires prioritizing surveillance in under-studied regions and standardizing methodologies. Furthermore, region-specific control strategies must be developed using a “One Health” approach, one that integrates surveillance and stewardship interventions across human healthcare, agricultural, and environmental sectors.

## Introduction

Antimicrobial resistance (AMR) is a major global public health threat with a disproportionate impact on Western Sub-Saharan Africa. While AMR encompasses resistance to drugs targeting bacteria, viruses, fungi, and parasites, the health crisis is largely driven by antibiotic resistance (ABR) in bacteria. In Ghana, ABR caused an estimated 5,900 direct deaths and contributed to 25,300 associated deaths in 2019, surpassing the mortality from many other public health challenges (Donkor et al., 2024; Kariuki et al., 2022; Donkor and Newman, 2011; Agyepong et al., 2018). The ESKAPE pathogens (*E. faecium*, *S. aureus*, *K. pneumoniae*, *A. baumannii*, *P. aeruginosa*, and *Enterobacter* species) are a significant driver of this burden (Agyepong et al., 2018; Koduah et al., 2021). Given its overwhelming clinical prevalence and shared resistance epidemiology with other Enterobacteriaceae in Ghana, this review expands its scope to include *Escherichia coli*, effectively analyzing the “ESKAPEE” group.

In response to this broad threat, Ghana established its five-year National Action Plan (NAP) on Antimicrobial Resistance (AMR) in 2017 (Koduah et al., 2021; Opintan, 2018), an integrated approach that acknowledges that the health of people is closely connected to the health of animals and our shared environment. However, the effectiveness of a national strategy depends on understanding sub-national dynamics, as ABR patterns are not uniform (Koduah et al., 2021; Opintan et al., 2015; Walana et al., 2023; Labi et al., 2021; Dekker et al., 2016). For instance, a region with intensive poultry farming may face different resistance challenges than a densely populated urban center with sanitation issues, requiring tailored interventions. Regional heterogeneity driven by variations in pathogen prevalence, local prescribing habits, healthcare infrastructure, and agricultural practices can significantly influence the success of interventions (Agyepong et al., 2018; Opintan et al., 2015; Afriyie et al., 2020). Despite this, a comprehensive assessment of regional ABR variations in Ghana remains largely absent from the literature. Existing data are often fragmented, geographically concentrated in a few urban centers, and methodologically inconsistent, preventing the formation of a cohesive national ABR map (Iskandar et al., 2021; Labi et al., 2019; Storr et al., 2017).

This narrative review aims to address this evidence gap by systematically collating and synthesizing the available literature on ESKAPEE pathogens across Ghana. Our primary objectives are to: (1) map the geographic distribution and intensity of existing ABR research to identify surveillance hotspots and critical data gaps; (2) synthesize published data to describe regional variations in the prevalence of ESKAPEE pathogens and their reported resistance profiles; and (3) collate findings on the regional distribution of key genetic resistance mechanisms documented in the literature. By clarifying these distinct patterns of heterogeneity from the available evidence, we provide a foundation for developing more targeted, region-specific policies and prioritizing future research.

## Methods

### Search strategy and eligibility criteria

A systematic search of four online databases (PubMed, Scopus, Web of Science, and African Journals Online) was conducted to identify relevant literature on ESKAPEE pathogens in Ghana. The search included all articles published from 2016 to 2025, a timeframe chosen to align with the development and implementation of Ghana’s first National Action Plan (NAP) on AMR (2017–2021) (Donkor et al., 2024; Jimah and Ogunseitan, 2020). Studies from this period are essential for reflecting the plan’s impact and highlighting subsequent progress and gaps. Search terms included combinations of “antimicrobial resistance,” “ESKAPEE,” “ESKAPE,” and the names of individual ESKAPEE pathogens, and “Ghana.”

Studies were included if they met the following criteria: (1) were original research articles; (2) reported primary data on at least one of the ESKAPEE pathogens isolated from clinical, animal, or environmental sources within Ghana; and (3) provided data on pathogen prevalence or antimicrobial susceptibility. Reviews, editorials, commentaries, and case reports were excluded. Two reviewers independently screened titles and abstracts, followed by a full-text assessment of potentially relevant articles to determine final eligibility.

### Study selection

**Figure 1:**
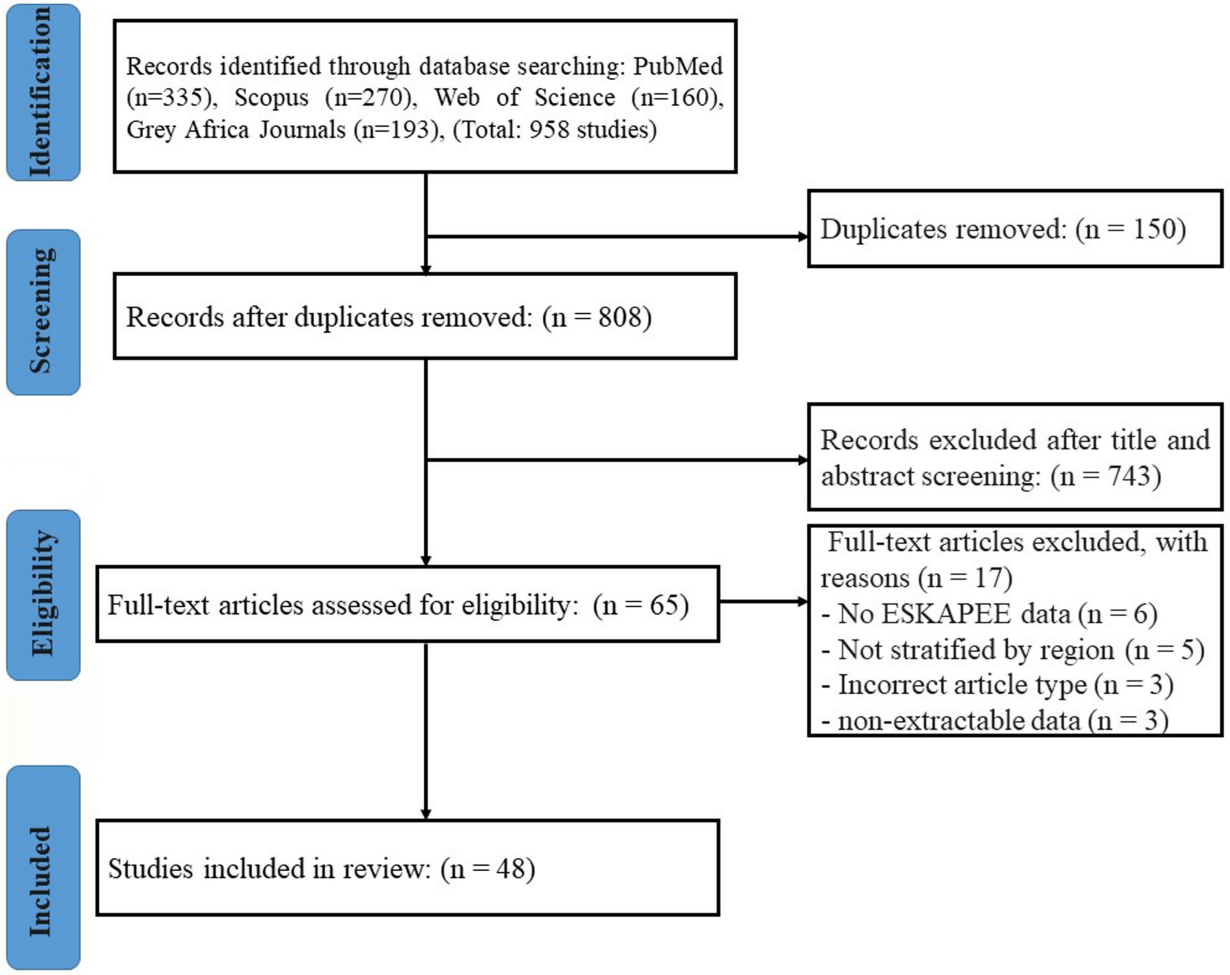
PRISMA flow diagram of the study selection process. The diagram illustrates the number of records identified, screened, assessed for eligibility, and included in the final review.

The initial database search yielded 958 publications, which was reduced to 808 after the removal of 150 duplicates (Figure 1). Following title and abstract screening, 743 articles were excluded. The full texts of the remaining 65 articles were assessed for eligibility, from which a final 48 studies were included in the narrative synthesis (Agyepong et al., 2018; Dekker et al., 2016; Bekoe et al., 2022; Saba et al., 2017; Boamah et al., 2017; Quansah et al., 2019; Dwomoh et al., 2022; Eibach et al., 2018; Calland et al., 2023; Gnimatin et al., 2022; Krumkamp et al., 2020; Janssen et al., 2018; Mohammed et al., 2018; Donkor et al., 2023; Codjoe et al., 2019; Asafo-Adjei et al., 2018; Sampah et al., 2023; Obeng-Nkrumah et al., 2024; Ohene Larbi et al., 2022; Karikari et al., 2022; Osei et al., 2022; Owusu et al., 2023; Odonkor et al., 2022; Adomako et al., 2021; Ahmed et al., 2022; Addae-Nuku et al., 2022; Acolatse et al., 2022; Inusah et al., 2021; Sampane-Donkor et al., 2017; Deininger et al., 2022; Asare et al., 2022; Omenako et al., 2022; Asamoah et al., 2022; Sah and Feglo, 2022; Vicar et al., 2023; Dsani et al., 2020; Baah et al., 2022; Asare Yeboah et al., 2024; Tettey et al., 2024; Afum et al., 2022; Labi et al., 2015; Andoh et al., 2017; Deku et al., 2021; Abana et al., 2019). The most common reasons for exclusion at the full-text stage were a lack of primary data (e.g., the article was a review, editorial, or commentary) and the absence of specific, usable data on either pathogen prevalence or antimicrobial susceptibility for the ESKAPEE pathogens. A summary of the characteristics and key data extracted from each of these included studies is presented in Supplementary Table S1.

### Study characteristics

The 48 selected publications included observational studies comprising a majority of cross-sectional designs (Sampah et al., 2023; Karikari et al., 2022), several retrospective analyses (Agyepong et al., 2018), and at least one longitudinal study (Addae-Nuku et al., 2022). Overall, these studies fall under the umbrella of “One Health,” as they included isolates sourced from human clinical settings, animals, and the environment. Some studies were comprehensive, investigating multiple ESKAPEE pathogens across different sources (Calland et al., 2023), while many focused on a single pathogen or setting (Odonkor et al., 2022).

Methodologies for bacterial identification varied, with many studies relying on traditional culture-based and biochemical methods (Asamoah et al., 2022), while others employed more advanced techniques like MALDI-TOF Mass Spectrometry (Calland et al., 2023; Asare Yeboah et al., 2024) and whole genome sequencing (WGS) for in-depth characterization (Dekker et al., 2016; Tettey et al., 2024). Antimicrobial susceptibility was most often determined using the Kirby–Bauer disk diffusion method, with a smaller number of studies using automated systems like Vitek-2 or determining minimum inhibitory concentrations (MICs). Interpretation of results was predominantly based on CLSI or EUCAST guidelines.

### Data extraction

Data from each of the 48 included studies were extracted independently by two reviewers using a standardized form (Supplementary Table S1). This process involved collecting study characteristics, such as the first author, publication year, geographical region(s), study design, and sample source (human clinical, animal, or environmental). Detailed laboratory information was also recorded, covering the methods for bacterial identification (e.g., culture-based, Vitek-2), antimicrobial susceptibility testing (AST) (e.g., Kirby–Bauer disk diffusion), and the interpretation guidelines used (CLSI, EUCAST). Key outcome data on pathogen prevalence, antimicrobial resistance rates, and specific genetic resistance mechanisms (e.g., *blaCTX-M-15*, *blaNDM-1*) were systematically collated.

### Risk of bias assessment

The methodological quality of the included observational studies was assessed using the ROBINS-I (Risk of Bias in Non-Randomized Studies-of Interventions) tool and visualized using the Robvis web app^1^ (McGuinness and Higgins, 2021). This tool provides a structured framework for evaluating study integrity by assessing potential bias across seven key domains. For this review, the most critical domains included bias due to confounding factors, the selection of study participants, the measurement of outcomes, and the selection of the reported result. Based on the evaluation across these domains, each study was assigned an overall judgment, which we categorized for this review as having a “low risk,” “some concerns,” or “high risk” of bias (Supplementary Figures S1, S2).

The assessment revealed that a majority of the studies (*n* = 35, 73%) were judged to have “some concerns” regarding bias, most frequently related to potential confounding factors and the selective reporting of results. Furthermore, 10 studies (21%) were rated as having a “high risk” of bias, often due to insufficient detail on the methods used for bacterial identification or antimicrobial susceptibility testing. Only 3 studies (6%) were deemed to have a “low risk” of bias, meeting all criteria for methodological rigor. These findings underscore the significant heterogeneity in study quality across the available literature on ABR in Ghana.

### Data synthesis

Given the significant heterogeneity in the methodologies of the included studies, a quantitative meta-analysis was not possible. Instead, a narrative synthesis was performed. The extracted data on pathogen prevalence, resistance profiles, and resistance mechanisms were collated, organized by geographical region, and summarized thematically. This approach allowed for the mapping of ABR patterns and the identification of regional differences and research gaps. To assess the relationship between research output and demographic factors, a Pearson correlation analysis was conducted to compare the number of studies per region with the regional population data from the 2021 Ghana Census.

## Results

### Geographic distribution of ABR research

**Figure 2:**
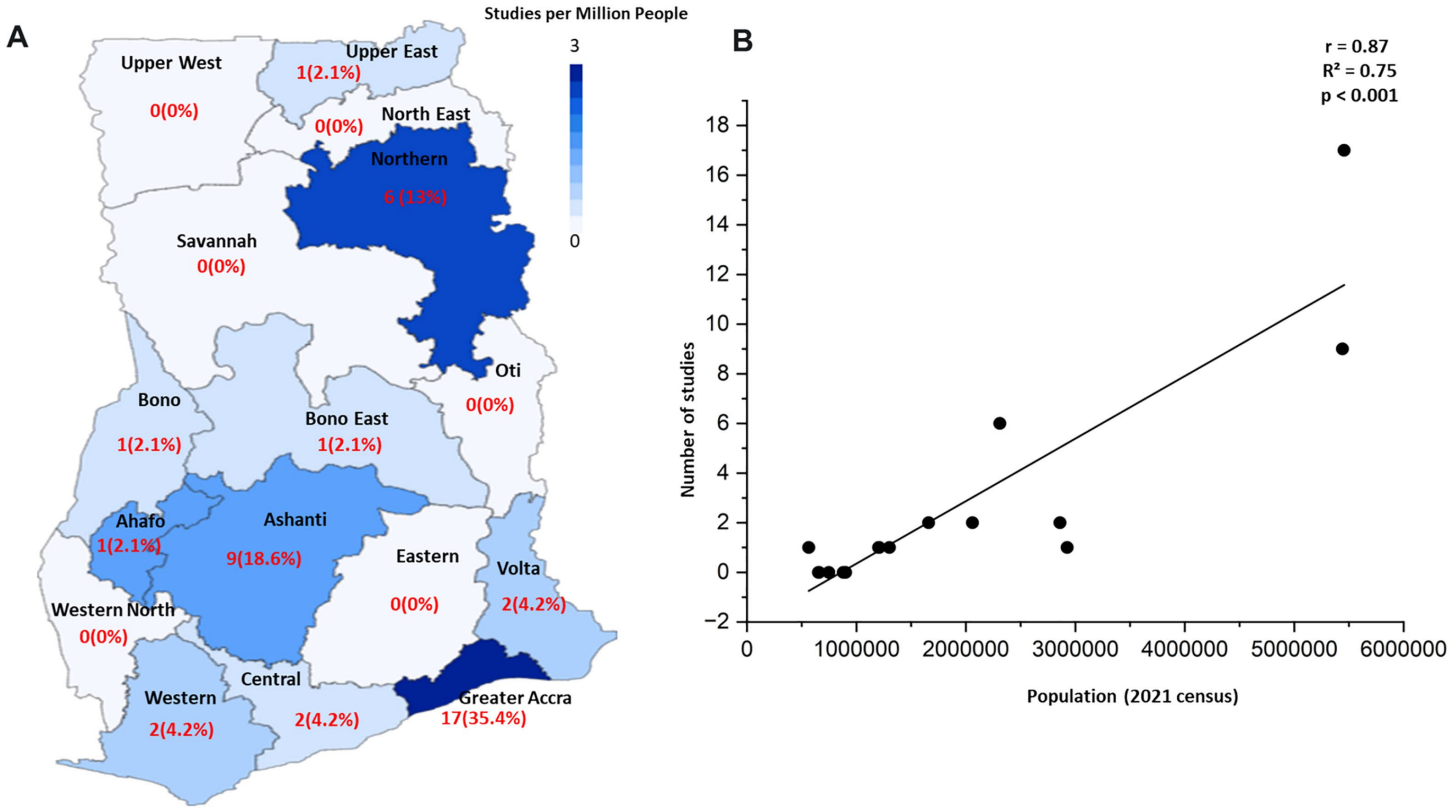
Geographic distribution and population correlation of ABR research in Ghana. **(A)** A choropleth map showing the intensity of ABR research across Ghana’s 16 administrative regions. Regions are shaded based on the number of studies per one million people (using 2021 census data), with darker shades indicating higher research intensity. The raw count of studies and its percentage of the total are labeled within each region. **(B)** A scatter plot showing a highly significant positive correlation between the regional population size and the number of studies conducted (Pearson’s *r* = 0.87, *R*^2^ = 0.75, *p* < 0.001). The trend line indicates that research intensity is higher in more populous areas. The plot also highlights key outliers, such as the Central and Eastern regions, which are populous but have fewer studies than predicted by the trend, identifying them as under-studied “blind spots”.

The 48 included studies show a pronounced geographical disparity in research focus across Ghana’s 16 administrative regions (Table 1 and Figure 2). A simple count reveals that research is heavily concentrated in three regions: Greater Accra (*n* = 17), Ashanti (*n* = 9), and Northern region (*n* = 6). When normalizing research output by regional population, we observed high heterogeneity in the research intensity across regions (Figure 2A). Notably, the Northern region has a high research intensity relative to its population (3 studies per million people), comparable to the Greater Accra region (3 studies per million). Moreover, we found a significant positive correlation between regional population size and the number of studies conducted (Figure 2B, Pearson’s *r* = 0.87, *R*^2^ = 0.75, *p* < 0.001), indicating that research efforts are concentrated in more populous regions. However, analyzing the deviations from this trend allows for the identification of regions that are outliers. For example, the Central and Eastern regions have large populations but a disproportionately low number of studies, marking them as significantly under-researched relative to their population size.

**Table 1:** Geographic coverage of antibiotic resistance research in Ghana.

Region	Number of studies	Percentage of total (%)
Greater Accra region	17	35.4
Ashanti region	9	18.8
Northern region	6	12.5
Central region	2	4.2
Eastern region	1	2.1
Upper East region	1	2.1
Bono East region	1	2.1
Ahafo region	1	2.1
8 other regions^*^	0	0
Multicenter	10	20.8
Total included	48	100

^*^Upper West, Bono, Oti, Savannah, North East, Western North, Western, Volta regions.

### Regional prevalence and resistance of ESKAPEE pathogens

Analysis of the collated data revealed significant regional heterogeneity in both the prevalence of ESKAPEE pathogens and their resistance profiles. While methodological differences across studies precluded a formal statistical meta-analysis, the narrative synthesis clearly shows distinct regional patterns. For example, the reported prevalence for *E. coli* in clinical samples in the highly urbanized Greater Accra region (up to 46%) was substantially higher than in the Northern region (up to 16%), suggesting potential differences in pathogen distribution between regions. *E. coli* and *K. pneumoniae* were consistently among the most prevalent Gram-negative pathogens identified in clinical samples across most regions (Table 2). For instance, prevalence for *E. coli* and *K. pneumoniae* was reported as high as 46 and 26% in Greater Accra region, respectively, while *K. pneumoniae* was a leading cause of infections in the Northern region with a prevalence of up to 27%.

**Table 2:** Regional prevalence of key ESKAPEE pathogens in Ghana (%).^*^

Pathogen /Region	*E. faecium*	*S. aureus*	*K. pneumoniae*	*A. baumannii*	*P. aeruginosa*	*Enterobacter* spp.	*E. coli*
Accra region	NR	NR	11.2–25.5	12.0	5.4–7.5	10.9–15.0	26.4–46.0
Ashanti region	NR	NR	5.0	NR	NR	53.8	NR
Northern region	NR	23.0	9.1–27.0	NR	19.7	NR	9.1–16.0
Central region	NR	NR	9.1	NR	NR	NR	15.8–40.9

^*^Prevalence ranges (%) are collated from the 48 included studies. Ranges are shown where multiple studies reported data. Only regions with more than one study were included. NR, not reported in the included studies from this region.

A high burden of antibiotic resistance was evident across all studied pathogens, with specific patterns varying by region (Table 3). Resistance to ampicillin and third-generation cephalosporins (e.g., ceftriaxone) was particularly alarming, frequently exceeding 70% for *E. coli* and *K. pneumoniae* in major urban centers. Notably, the Ashanti region reported extensively drug-resistant *A. baumannii* and *P. aeruginosa*, with both pathogens showing 100% multidrug resistance (MDR) in one hospital-based study. The prevalence of extended-spectrum beta-lactamase (ESBL) production was also a major concern, reaching 49.1% among *Enterobacteriaceae* in the Central region.

**Table 3:** Antibiotic resistance rates of ESKAPEE pathogens in selected regions (%).

Region	Pathogen	Ampicillin	Ceftriaxone	Ciprofloxacin	Meropenem	MDR	ESBL
Greater Accra region	*E. coli*	97.2	73.7	55.3	5.6	94.3	44.6
	*K. pneumoniae*	100	77.8	73.8	5.6	96.4	44.6
	*A. baumannii*	—	56.3	55.3	90	97.5	—
	*P. aeruginosa*	—	56.3	55.3	52	100	—
Ashanti region	*Acinetobacter* spp.	—	—	—	Low	100^a^	—
	*P. aeruginosa*	—	—	—	Low	100^a^	—
Northern region	*E. coli*	100	50	100	Low	87.2	—
	*K. pneumoniae*	97	53.3	73.8	Low	84.2	—
Central region	*E. coli*	74.3	58.3	-	5.7	35.2	49.1
	*S. aureus*	100	90.8	20.5	86.7	—	—
Bono East region	*Staphylococcus* spp.	100	90.8	20.5	High	15.5	—

^a^MDR status was determined based on resistance to additional antibiotic classes tested in the original study but not displayed in these specific columns.

### Distribution of key resistance mechanisms

**Figure 3:**
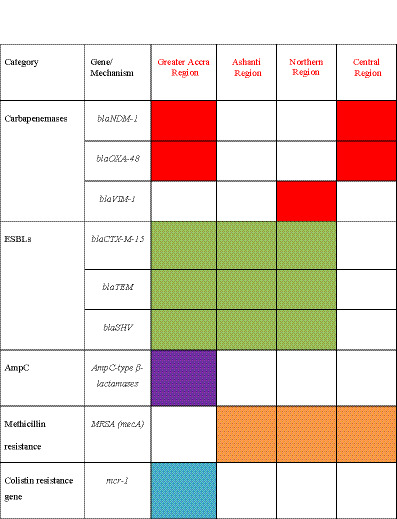
Presence/absence matrix of key resistance mechanisms in Ghana. The matrix shows the reported presence of specific resistance genes and mechanisms across four major regions, based on the literature reviewed. A colored cell indicates that the mechanism was reported in at least one study from that region, while a blank cell indicates that it was not reported. The colors depict the different categories of antibiotic resistance mechanisms.

The genetic drivers of resistance also showed notable regional variation, with specific findings from each study detailed in Figure 3 and Supplementary Table S1. The ESBL gene *bla*CTX-M-15 was identified across multiple regions, including Greater Accra, Ashanti, and Northern regions, confirming its role as a dominant resistance driver in Ghana. Critically, several classes of carbapenemase genes have emerged, with *bla*NDM-1 and *bla*OXA-48 reported in the Greater Accra and Central regions (Eibach et al., 2018; Obeng-Nkrumah et al., 2024; Agyepong, 2016; Quansah et al., 2019; Dwomoh et al., 2022). The *blaVIM-1* gene was also reported in a multi-center study that included Northern Ghana (Codjoe et al., 2019), suggesting a distinct regional molecular epidemiology. The presence of methicillin-resistant *S. aureus* (MRSA), identified phenotypically or via the *mecA* gene, was noted in the Ashanti, Central, and Northern regions (Dekker et al., 2016; Krumkamp et al., 2020). Moreover, one study detected the plasmid-mediated colistin resistance gene, *mcr-1*, in *E. coli* from animal sources in the Greater Accra region (Ohene Larbi et al., 2022). Additionally, AmpC-type beta-lactamases were reported, primarily in studies from the Greater Accra region (Owusu et al., 2023). The potential drivers for these observed regional variations, including interconnected environmental, agricultural, and socioeconomic factors, are explored in detail in the Discussion section.

## Discussion

### Heterogeneity in research coverage: a map of hotspots and blind spots

This narrative review confirms that the ABR challenge in Ghana is not a uniform crisis but reflects a patterned landscape defined by at least three distinct dimensions of regional heterogeneity. The most striking finding is the profound disparity in ABR research and surveillance. Our analysis reveals that research is heavily concentrated in the Greater Accra (35.4%) and Ashanti (18.8%) regions (Table 1 and Figure 2). This focus on urbanized centers with major tertiary hospitals creates data “hotspots” but leaves vast areas of the country, such as the Upper West, Oti, and Savannah regions, as critical “blind spots.” This geographical bias means that national ABR policies are often informed by data that may not be representative of the challenges faced in more rural or less-resourced parts of the country.

Beneath these surveillance gaps, the available data show clear regional variations in the prevalence and resistance profiles of specific ESKAPEE pathogens. For instance, our synthesis shows that while some studies report *K. pneumoniae* as a leading cause of infections in the Northern region, others highlight extensively drug-resistant *A. baumannii* as a major threat in Ashanti region hospitals (Agyepong et al., 2018). Furthermore, the notable prevalence of MRSA in both human and agricultural contexts in the Central region points to a distinct “One Health” challenge that may be less pronounced elsewhere.

The most granular level of heterogeneity is seen in the underlying genetic drivers of resistance. This is best visualized in Figure 3, which, based on our synthesis, shows a clear regional clustering of key resistance genes. For instance, the aforementioned concentration of carbapenemase genes in southern urban regions (Figure 3) suggests that interventions there should focus on enhancing wastewater treatment and hospital infection control. In contrast, the evidence of zoonotic MRSA and *mcr-1* transmission in the Central and Greater Accra regions, respectively, implies that regional strategies there must place a stronger emphasis on veterinary surveillance and promoting responsible antibiotic use in agriculture.

### Drivers of heterogeneity and implications of research gaps

The patterns observed in this review suggest that the reported heterogeneities are driven by a complex interplay of factors. At a regional level, environmental risk factors such as waste management, agricultural practices, and sanitation are recognized as significant contributors to the spread of ABR across West Africa (Adenaya et al., 2025). Our review builds on this by demonstrating that the impact and prevalence of these drivers are not uniform within a single country. The concentration of research in the Greater Accra and Ashanti region directly correlates with the presence of major research universities (e.g., University of Ghana, KNUST) and tertiary referral hospitals with better-equipped laboratories. This creates a cycle where research capacity dictates data availability, leaving vast areas, particularly in northern Ghana, as critical “data blind spots.” Beyond infrastructure, this points to underlying limitations in logistical capacity, research funding allocation, and the distribution of scientific expertise across the country.

These research gaps have profound implications. The absence of surveillance data from entire regions means that local outbreaks of highly resistant pathogens could go undetected, leading to poor clinical outcomes and undermining public health security. This directly impacts our understanding of, and ability to address, regional health disparities. National policies or treatment guidelines based on data skewed toward urban southern centers may be ineffective or even counterproductive in regions where the pathogen distribution and resistance mechanisms are different.

To address these gaps, targeted capacity-building is essential. We propose that potential collaborations could be fostered between Ghana’s major universities (e.g., University of Ghana, KNUST) and regional centers of excellence, such as the West African Centre for Cell Biology of Infectious Pathogens (WACCBIP), in partnership with international bodies like the Africa CDC. Such partnerships could create a “hub-and-spoke” model to extend diagnostic and research capabilities into the under-served regions.

Furthermore, innovative and cost-effective surveillance methods employed in other settings could be adapted for Ghana. For example, wastewater-based epidemiology offers a powerful tool for monitoring ABR trends at a community level, providing a non-invasive snapshot of the resistance genes circulating in a population, including those in “blind spot” regions (Clarke et al., 2024; Balcázar, 2025; Foxman et al., 2025). This, combined with targeted genomic surveillance of clinical isolates, would provide a far more comprehensive and efficient framework for mapping and responding to the ABR threat.

### Limitations of the evidence

Several limitations should be considered when interpreting the findings of this review. Firstly, the significant methodological heterogeneity across the primary studies including differences in study design, laboratory methods, and reporting standards as detailed in Supplementary Table S1 precluded a quantitative meta-analysis and posed challenges for direct comparison. Secondly, as our risk of bias assessment reveals, a notable portion of the included literature was assessed as having “some” or a “high risk” of bias, particularly related to the selective reporting of results. This suggests that studies reporting high or novel resistance rates may be more likely to be published, potentially overestimating the ABR burden in some areas. Finally, the most significant limitation is the incomplete nature of the national ABR “map” that this review was able to construct. The heavy research concentration in a few urban centers means that vast regions remain critical surveillance gaps. The absence of data from these areas does not imply the absence of ABR; rather, it highlights an urgent need for targeted research to understand the full scope of the problem in Ghana.

## Conclusion and recommendations

### Summary of evidence: a nuanced view of a national crisis

This narrative review presents a nuanced picture of ABR in Ghana, confirming it as both a widespread national crisis and a regionally patterned challenge. The crisis is evident in the homogeneously high rates of multidrug resistance to common antibiotics, a consistent finding across all studied regions as shown in Table 3. The patterned nature, however, is revealed in the heterogeneity of its underlying components: the specific distribution of ESKAPEE pathogens, the distinct regional clustering of critical resistance genes like carbapenemases (e.g., *bla_NDM-1*, *bla_OXA-48*, and *bla_VIM-1*), and the varying influence of local One Health drivers. Therefore, while the ABR burden is undeniably national in scale, tackling it effectively requires a nuanced, region-specific approach that addresses this complex landscape.

### Recommendations for future research to refine the AMR map

The challenge of mapping sub-national ABR heterogeneity is not unique to Ghana. The approach outlined in this review, synthesizing fragmented data to identify hotspots and blind spots provides a valuable framework for other nations. The following research priorities are therefore essential for refining the ABR map in Ghana and can serve as a guide for other low- and middle-income countries (LMICs):

- Prioritize surveillance in underrepresented regions: Target funding and research efforts to establish baseline ABR data in regions with minimal or no existing information. In Ghana, this includes the Upper West, Bono, Oti, Savannah, and other regions identified as “blind spots” in our analysis.
- Standardize methodologies for data collection and reporting: Promote adherence to internationally recognized guidelines (e.g., CLSI, EUCAST) for bacterial identification, antimicrobial susceptibility testing, and resistance gene characterization to improve data comparability and enable robust meta-analyses.
- Conduct integrated “One Health” research: Implement systematic research programs that investigate the prevalence and transmission dynamics of ABR across human, animal, and environmental interfaces within specific regions, including genomic epidemiology to track the flow of resistance genes.
- Investigate socioeconomic and behavioral drivers: Employ qualitative and quantitative research methods to understand how local factors such as healthcare access, antibiotic prescribing habits, public awareness, and agricultural practices contribute to regional ABR patterns.

### Recommendations for policy and practice

The central finding of this review that national ABR crises are composed of distinct regional patterns has profound implications for policy. The following recommendations offer a strategic framework for translating surveillance data into targeted, effective policy and practice:

- Develop and implement region-specific ABR control strategies: Evolve the National Action Plan to include detailed, tailored strategies for different regions, addressing their unique pathogen profiles, dominant resistance mechanisms, and underlying drivers.
- Strengthen laboratory capacity equitably across all regions: Invest in infrastructure, equipment, and training for microbiology laboratories, especially in under-resourced districts, to ensure accurate and consistent ABR diagnostics and surveillance.
- Tailor antimicrobial stewardship programs to local resistance patterns: Regularly update clinical guidelines based on regional surveillance data to promote the rational use of antibiotics and preserve the efficacy of last-resort treatments.
- Design and implement targeted public health campaigns: Create educational initiatives on responsible antibiotic use and hygiene that are designed with specific regional contexts, languages, and cultural norms in mind to maximize their impact.

## Footnotes

1
https://www.riskofbias.info/welcome
