# Supplementary material for: Mapping antibiotic resistance in Ghana: a narrative review of regional variations in antibiotic-resistant ESKAPEE pathogens
Source: Front Microbiol. 2026 Jan 6;16:1696696. doi: 10.3389/fmicb.2025.1696696 (PMC12816315; doi:10.3389/fmicb.2025.1696696)
Supplement: Supplementary file 2 [file Data_Sheet_1.pdf]

Supplementary Material 1: Risk of Bias Assessment

The methodological quality of the 48 included observational studies was assessed using a framework adapted from the ROBINS-I (Risk Of Bias In Non-randomized Studies - of Interventions) tool. Given that the included studies are primarily descriptive and non-interventional, the ROBINS-I domains were adapted to evaluate the key potential sources of bias in AMR surveillance research as follows:

Domains Assessed:

1. **Bias due to Confounding:** Evaluated the potential for uncontrolled factors to influence the results.
2. **Bias in Selection of Participants:** Assessed whether the sampling method was likely to yield a representative sample.
3. **Bias in Measurement of Outcomes:** Critically evaluated the methods for bacterial identification and antimicrobial susceptibility testing (AST). This was considered a key domain.
4. **Bias in Selection of the Reported Result:** Assessed the likelihood that authors selectively reported significant findings as shown in supplementary figure 1 (S1).

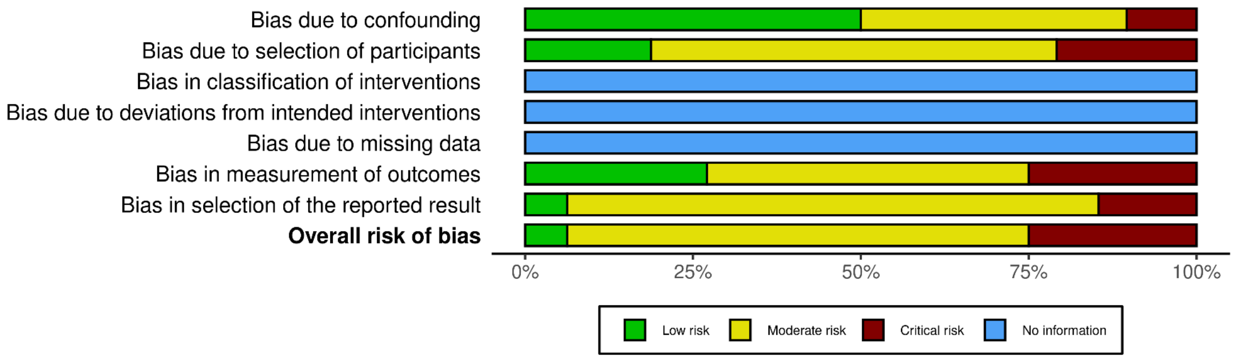

Figure S1: Risk of Bias Assessment. Summary of risk of bias for each domain across all included studies.

The analysis of the 48 studies resulted in the following distribution: **Low Risk of Bias: 3 studies (6%)**; **Moderate Risk (Some Concerns): 35 studies (73%)**; and **Critical Risk (High Risk): 10 studies (21%)**. Detailed risk of bias judgments for each domain for each individual study is shown in figure S2.

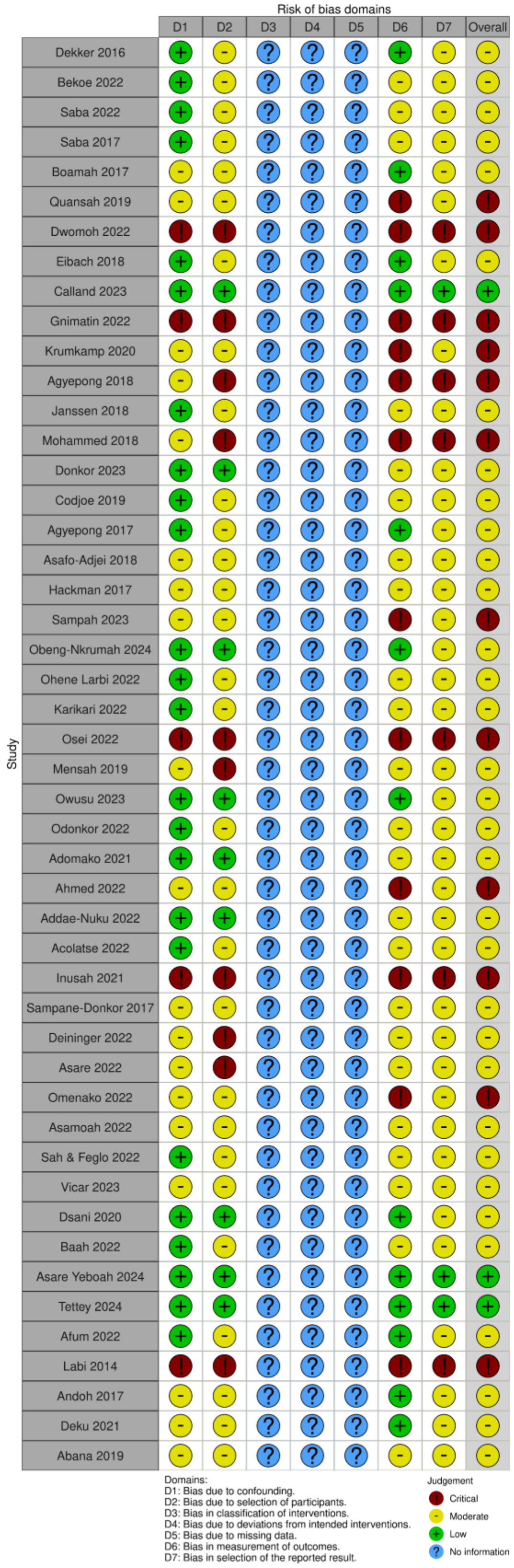

Figure S2: Risk of Bias Assessment. Detailed risk of bias judgments for each domain for each individual study.

Judgment Criteria:

- **Low Risk:** The study demonstrated high methodological rigor (e.g., used robust identification methods like MALDI-TOF/WGS, specified CLSI/EUCAST guidelines, and used a systematic sampling method).
- **Moderate Risk (Some Concerns):** The study was methodologically sound but had weaknesses that could introduce bias (e.g., used standard methods but lacked detail on interpretation guidelines or sampling methods).
- **Critical Risk (High Risk):** The study had significant methodological flaws, typically due to missing critical information on identification or AST methods, making the results difficult to validate.
